# hPDB – Haskell library for processing atomic biomolecular structures in protein data bank format

**DOI:** 10.1186/1756-0500-6-483

**Published:** 2013-11-23

**Authors:** Michał Jan Gajda

**Affiliations:** 1 NMR-2, Max Planck Institute for Biophysical Chemistry, Am Faßberg 11, Göttingen, Germany

**Keywords:** Structural biology, Protein DataBank file format, Parallel parser, Parser efficiency, Column-based parsing

## Abstract

**Background:**

Protein DataBank file format is used for the majority of biomolecular data available today. Haskell is a lazy functional language that enjoys a high-level class-based type system, a growing collection of useful libraries and a reputation for efficiency.

**Findings:**

I present a fast library for processing biomolecular data in the Protein Data Bank format. I present benchmarks indicating that this library is faster than other frequently used Protein Data Bank parsing programs. The proposed library also features a convenient iterator mechanism, and a simple API modeled after BioPython.

**Conclusion:**

I set a new standard for convenience and efficiency of Protein Data Bank processing in a Haskell library, and release it to open source.

## Findings

### Background

The Protein Data Bank (PDB) is a widely used data repository of atomic resolution, three-dimensional protein and nucleic acid structures [1]. The rapid growth of structural data enables key endeavors to bring knowledge of genomes [2] to the structure and function of large biomolecules. In addition to sequence searches and genome assemblies, efficient and reliable structural data processing are one of the most important and common structural bioinformatics tasks [3].

Haskell is a modern, lazy, pure functional language [4,5] that enjoys fluid syntax, and clarity comparable to Python [6], as well as an efficient compiler that often generates code approaching the speeds of industry standard languages such as C [7] or C++ [8].

### Library interface

The library is a comprehensive solution for the parsing, rapid processing and writing of PDB files. I introduce the library by providing examples and describing the underlying data structures^a^, and finally, I present an evaluation of its efficiency.

#### Simple use example

A parser example^b^ – a script that reads a structure containing multiple models and splits the structure into single models. 

Here, I extract a list of models from a **Bio.PDB.Structure.Structure**^c^ object (V.toList.**models**^d^), and repackage each model as a separate structure. These structures are then written using **PDB.write**.

I use Data.Vector.Vector to store all lists, including list of models, within the library. Data.Vector.Vector significantly reduces memory use after reading PDB files (see the Benchmarks section.)

To complete the program I include a command line interface: 

A simple **PDB.parse** action returns a structure, to which I apply **splitModels** and zip a list of results with model numbers. These results are used to generate names of output files, that are then written using the **PDB.write** IO action. ByteString is used rather than [Char] within the library for everything except file paths (FilePath), due to efficiency considerations^e^.

#### Data structure describing molecules

**Figure 1:**
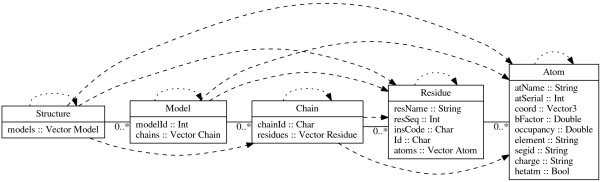
**Data structures used to describe PDB structures.** This figure represents a hierarchy of collections contained in each PDB **Structure**. Arrows represent the hierarchy of **Iterable** instances linking contained objects. Line style distinguishes between direct (continuous line) and transitive (dashed line) instances of **Iterable** class. For ease of use, I also allow to iterate over each type as a singleton collection (dotted line.) Below each datatype name I enumerate record components and their types for easy reference.

Different levels of collection hierarchy can be seen on Figure 1:

**Structure** that contains information about whole PDB entry; **Model** that shows a single model of the molecule; **Chain** describing a single polymer chain; **Residue** for a single monomer (aminoacid residue or nucleic acid base) within the polymer; **Atom** for a single atom location.

Names of these types correspond to the names used by PDB file format definition [1]. Those atoms which may have multiple locations within the model are described by several records, and those residues that have alternative mutants are also described by different records in accord with current practice of PDB [1].

#### Iterating with **Iterable**

For the different types of objects, I devised a custom **Iterable** class that allows iteration over all objects of each of many types contained within an argument object. This class generalizes map, and foldr iteration patterns used over lists, to hierarchical containers, and allows iteration over any type of contained objects, potentially in an indirect manner: 

The **itmap** method allows mutation of any of the objects of a given type *b* contained within type *a*. To compute a statistic over all contained objects **itfoldr** or **itfoldl** can be used.

The class **Iterable**a b may thus be viewed as a generalization of **Functor**, **Foldable**, and **Traversable** to *hierarchical collections* that may contain more than one class of objects. All Iterable instances, and method types are shown in Figure 1.

It is often convenient to use a monadic state when computing a statistic, or when renumbering residues. For this purpose, I use monadic variants of the following methods: 

For efficiency I introduce a rapid **itlength** method, and a strict **itfoldl** variant: 

Note that **itlength** is the only method using a *dummy* first argument to indicate the type of contained object to be counted. As all other methods use a function argument, automatic type inference finds the proper class instance without requiring a type declaration, as shown in the examples below^f^.

#### Structure analysis example

In the following examples I skip the command line interface, assuming that all functions input a parsed **Structure** object.

The most convenient interface for a complex cascade of container types within a PDB structure is composition based on fold, and map analogs.

To compute the center of mass of all **Atom** objects contained within a structure, I use a two pass algorithm: 

Here I use **itfoldl’** instantiated to the following type, automatically inferred from the types of addCoord and s: 

I will generalize this type to other types within the structure, when showing class **Iterable**. This generalization allows a function to have a type showing that it can take any object that contains **Atom** objects within: 

Then, I can subtract the computed center from all atomic coordinates with the **itmap** method, analogous to map for lists. In this example, mapping **PDB.Atom** objects within a **PDB.Structure**^g^, is used: 

This use of itmap has an instantiated type, that is automatically inferred from its first argument: 

#### Stateful modification

Simple **itmap**, and **itfoldl’** methods are not sufficient to perform a complex stateful operation such as renumbering residues starting from 1.

In this case, I use monadic analogs, such as **itmapM**^h^: 

Such a code requires separate application to each chain, because residue numbering begins anew with each chain: 

Assigning consecutive serial numbers is handled by a state monad [9], as described below: 

Renumbering atoms within each model is more involved, because the PDB data format [10] mandates that TER records^i^ are counted along with the atoms, and these records do not have direct representation in *hPDB* data structures. 

In this example renumberAtoms may be labelled with the monomorphic (and useful) type: 

Although the automatically inferred type allows this function to act not only on the entire **Structure** but also on any single **Model** that contains the **Chain** objects.

#### Example applications

hPDB’s speed and ease of use has allowed for rapid implementation of typical functions such as: orienting structure so that the longest diameter corresponds to the Y axis, and the second longest cross-sectional dimension corresponds to the X axis (CanonicalAxes in hPDB-examples package), normalizing PDB files for use by applications restrictive with respect to file format (CleanPDB), and examining the sequence of main polymer chain or geometric parameters of small-angle scattering shape reconstructions (Rg example) with minimal code.

### Results and discussion

#### Benchmarks

For the benchmark, *hPDB* was compiled in single-threaded and multi-threaded mode by GHC v7.6.2.

I benchmarked three other PDB parser libraries *BioJava*[11] (v3.0.5-2), *BioRuby*[12] (v1.4.2 using standard Ruby VM v1.9.3p194), and *BioPython*[13] (v1.60, using standard CPython 2.7.4 [6] implementation). I include time results for common molecular visualization programs (as these are required to show a complete structure quickly): *RasMol*[14] (v2.7.5.2), known for a long history of optimization and written in C; *PyMol*[15] (v1.5.0.1), written in both C and Python; and *Jmol*, written in Java [16] (v12.2.32). Each parser’s CPU time is reported in Table 1.

**Table 1:** Total CPU time in seconds

**PDB entry**	**hPDB par.**	**hPDB seq.**	**BioJava**^**1**^	**BioRuby**	**BioPython**	**PyMol**	**RasMol**	**Jmol**^**1**^
1CRN	≥0.01	≥0.01	0.38	0.03	0.31	0.06	0.06	1.96
3JYV	0.27	0.26	1.31	0.89	1.26	0.28	0.28	3.52
1HTQ	5.08	4.63	6.66	16.52	23.41	3.94	4.90	25.82

^1^Jmol and BioJava use multiple threads, thus completion time is closer to half the CPU time than to the sum of CPU time and I/O time (as indicated in Table 3).

In the case of libraries, I used operating system calls or ps program to determine the upper bounds of memory used in Table 2 (including purely virtual allocations).

**Table 2:** Total allocated memory in megabytes

**PDB entry**	**Input size**	**hPDB par.**	**hPDB seq.**	**BioRuby**	**BioJava**	**BioPython**
1CRN	49 kB	3	1	8	240	206
3JYV	5	41	35	85	302	324
1HTQ	76	609	547	1350	1180	2409

Haskell memory is reported for the current heap, in addition to the target space for copying garbage collector [17].

Note that *Jmol* and *BioJava* may both use more than one thread, which significantly reduces time-to-completion when using a multicore machine as reported in Table 3.

**Table 3:** Completion time after parsing in seconds

**PDB entry**	**hPDB par.**	**hPDB seq.**	**BioJava**	**BioRuby**	**BioPython**	**PyMol**^**2**^	**RasMol**^**2**^	**Jmol**^**2**^
1CRN	≥0.01	≥0.01	0.23	0.04	0.32	0.14	0.77	2.26
3JYV	0.09	0.28	0.71	0.94	1.43	0.38	0.86	2.81
1HTQ	1.39	4.79	3.24	17.14	24.01	4.22	5.73	12.86

^2^Includes the time needed for startup and closing the window.

The benchmarks were measured on a quad-core Intel®; Core™ i7 2600 processor running at 3.4 GHz^j^, 16 GB of 1333 MHz memory, and a SAMSUNG 470 Series solid-state disk. The system was running a 64-bit Ubuntu 13.04 with a standard Linux kernel package 3.8.0-31.

While *hPDB* may be expected to stand out in runtime comparisons to the bytecode-based dynamic language libraries *BioRuby* and *BioPython*, surprisingly, serial *hPDB* is faster than other parsers in compiled languages, with the exception of *PyMol*. The parallel version of the *hPDB* parser may be the fastest PDB parser on machines with at least 4 independent processing cores.

It was noted that memory use, even with a necessary overhead (2×) of Haskell’s copying garbage collector, compared favorably with memory used by other libraries.

Parsing the entire PDB archive (as of January 6th 2013, compressed, 16 GB) takes approximately 14.5 minutes using 4 cores in parallel, with total CPU and I/O time reported to be 50 minutes. No crashes are reported, but 8k lines (mostly meta data) are reported as erroneous^k^ because they are inconsistent with strict interpretation of PDB format [10].

Benchmarks show that in this specific application, the mildly optimized Haskell parser may provide speeds competitive with compiled languages such as Java and even lower level explicitly allocated languages such as C. Memory usage is also less than any other aforementioned library.

There is another Haskell library parsing PDB files on Hackage [18] called *PDBtools*, but it was not able to fully parse any of our example files because it does not handle errors in the read routine.

### Conclusions

I have shown clear uses of a nice high-level interface for the analysis and modification of molecule descriptions encoded in the PDB file format [10].

While there are many similar parsers written in other languages, this is the first one I am aware of in Haskell, that parses entire coordinate contents within the PDB repository. It is also efficient both in runtime and memory use, and thus, the preferable choice for sophisticated, high volume analyses.

While future work on analysis API extensions would likely further improve utility of this library, I believe that it is ready for production use, as indicated by the many code examples.

I conclude that in this specific application, Haskell has both ease of use and abstraction of high-level dynamic languages, along with a speed competitive with lower level explicit-allocation languages such as C.

## Availability and requirements

Source code is available as Additional files 1, 2 and 3 attached to the manuscript or from GitHub repository https://github.com/mgajda/hPDB, and released on Hackage as *hPDB*. It has been tested with several GHC versions including 7.0.3, 7.2.2, 7.4.2, and the recently released 7.6.2. It has few dependencies, and all are available from Hackage [18].

**Project name:** hPDB **Project home page:**http://hackage.haskell.org/package/hPDB**Source repositories:**http://github.com/mgajda/hPDBhttp://github.com/mgajda/hPDB-exampleshttp://github.com/mgajda/iterable**Operating system(s):** Platform independent **Programming language:** Haskell **Libraries:** Haskell Platform, AC-Vector **Other requirements:** GHC ≥ 7.0 **License:** BSD

## Endnotes

^a^ While this article contains only one figure showing the most important types for the API, two additional diagrams elucidating the library’s internal structure are available in the Additional files 4 and 5.

^b^ The command line interface for this function may be found in examples/SplitModels.hs in the *hPDB-examples* package.

^c^ Names defined in the hPDB package are emphasized in **bold** font for ease of reading. Other modules are the standard collection interface Data.Vector from the *vector* package, the 3D vector interface Data.Vector.V3 from the *AC-Vector* package, and Data.ByteString.Char8 from the *bytestring* package.

^d^ Note the use of Data.Vector for space efficient storage of data.

^e^ Most records in the PDB file format are ASCII-only; therefore, Unicode encoding is not necessary. As non-ASCII characters can only occur in comments and metadata, they may be decoded after parsing.

^f^ Type parameter *b* in declaration for itlength is a *dummy type argument* to specify the contained object types to be counted.

^g^ This declaration is less polymorphic than the actual **itmap** type, as demonstrated in the following section about **Iterable** class description.

^h^ Extended examples are present in the CleanPDB.hs example attached to the library.

^i^ Indicating termination of polymer chain, rather than an atom.

^j^ With overclocking switched off.

^k^ It is known that, after six different official releases of file format descriptions and many data remediation efforts, there is a small amount of data that does not entirely conform to the PDB archive format.

## Abbreviations

API: Application Programming Interface *(function and data declarations)*; ASCII: American Standard Code of Information Interchange *(7-bit text encoding)*; CPU: Central Processing Unit *(processor)*; I/O: Input/Output; PDB: Protein DataBank *(repository of biomolecular structural data)*; GHC: Glasgow Haskell Compiler; GWDG: Gesellschaft für Wissenschaftliche Datenverarbeitung mbH Göttingen – Göttingen Society for Scientific Data Processing.

## Competing interests

The author declares no competing interests.

## Supplementary Material

Additional file 1 **Source package archive.**hPDB.tgz contains full source distribution of the hPDB package. It is also available through the Hackage database at: http://hackage.haskell.org/package/hPDB. Click here for file

Additional file 2 **Source package archive.**hPDB-examples.tgz contains full source distribution of hPDB examples. It is also available through Hackage database at http://hackage.haskell.org/package/hPDB-examples. Click here for file

Additional file 3 **Source package archive.**iterable.tgz contains library definition of **Iterable** class, and macros helping in its instantiation written in Template Haskell [20]. It is also available through Hackage database at: http://hackage.haskell.org/package/iterable. Click here for file

Additional file 4 **Data type hierarchy.** Data type hierarchy showing all accessible types and data flow during parsing and printing. Hidden types are marked with *dotted* ellipses. Data flow is shown with *dashed* lines. Types marked with *solid ellipses* are part of API, and *solid lines* indicate direct type containment. Click here for file

Additional file 5 **Module diagram.**graphmod.svg contains a module diagram illustrating structure of hPDB. Click here for file
