# Supplementary figures and images for: hPDB – Haskell library for processing atomic biomolecular structures in protein data bank format
Source: BMC Res Notes. 2013 Nov 23;6:483. doi: 10.1186/1756-0500-6-483 (PMC3879085; doi:10.1186/1756-0500-6-483)

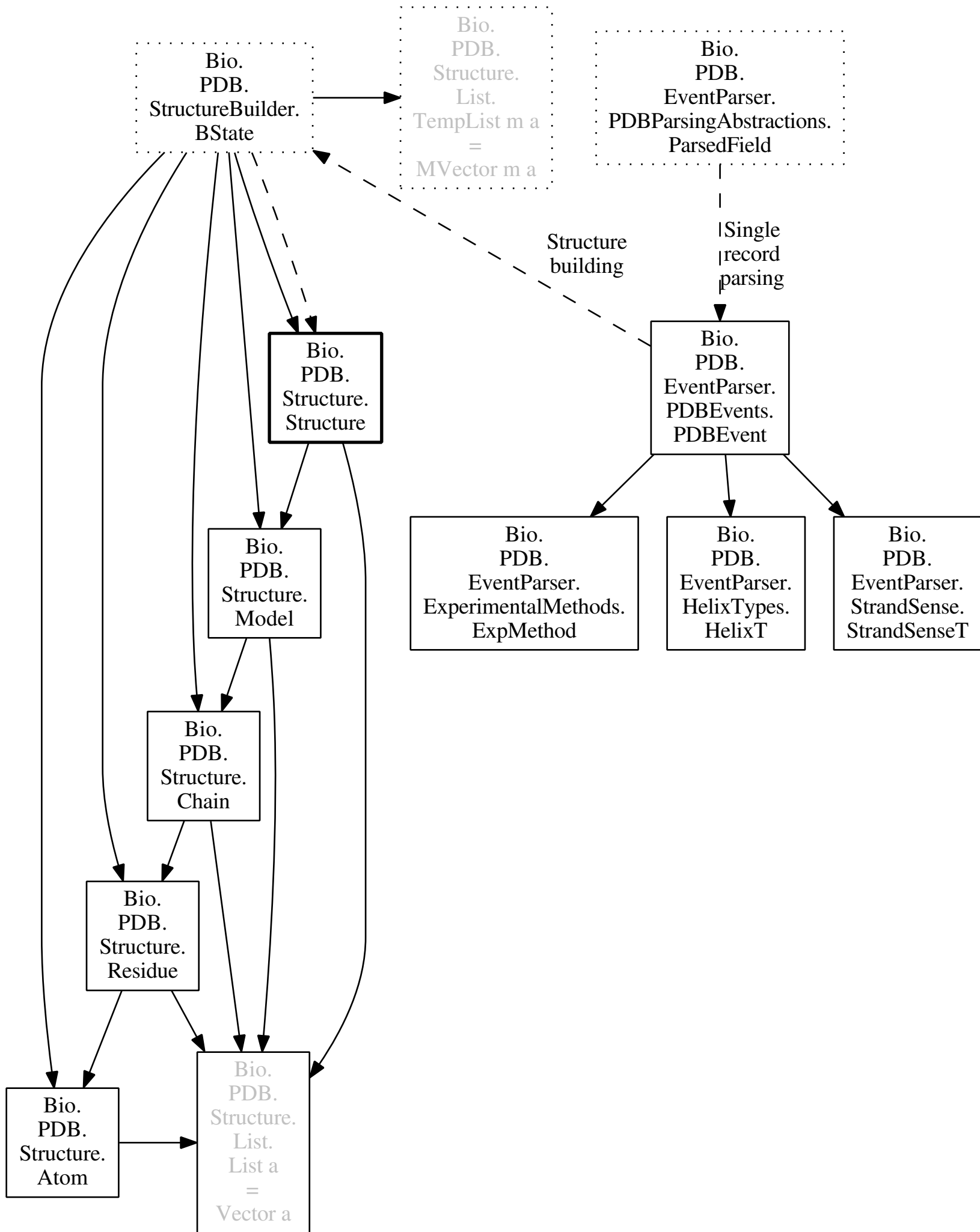

Supplement: Additional file 4 — Data type hierarchy. Data type hierarchy showing all accessible types and data flow during parsing and printing. Hidden types are marked with dotted ellipses. Data flow is shown with dashed lines. Types marked with solid ellipses are part of API, and solid lines indicate direct type containment. [file 1756-0500-6-483-S4.pdf]

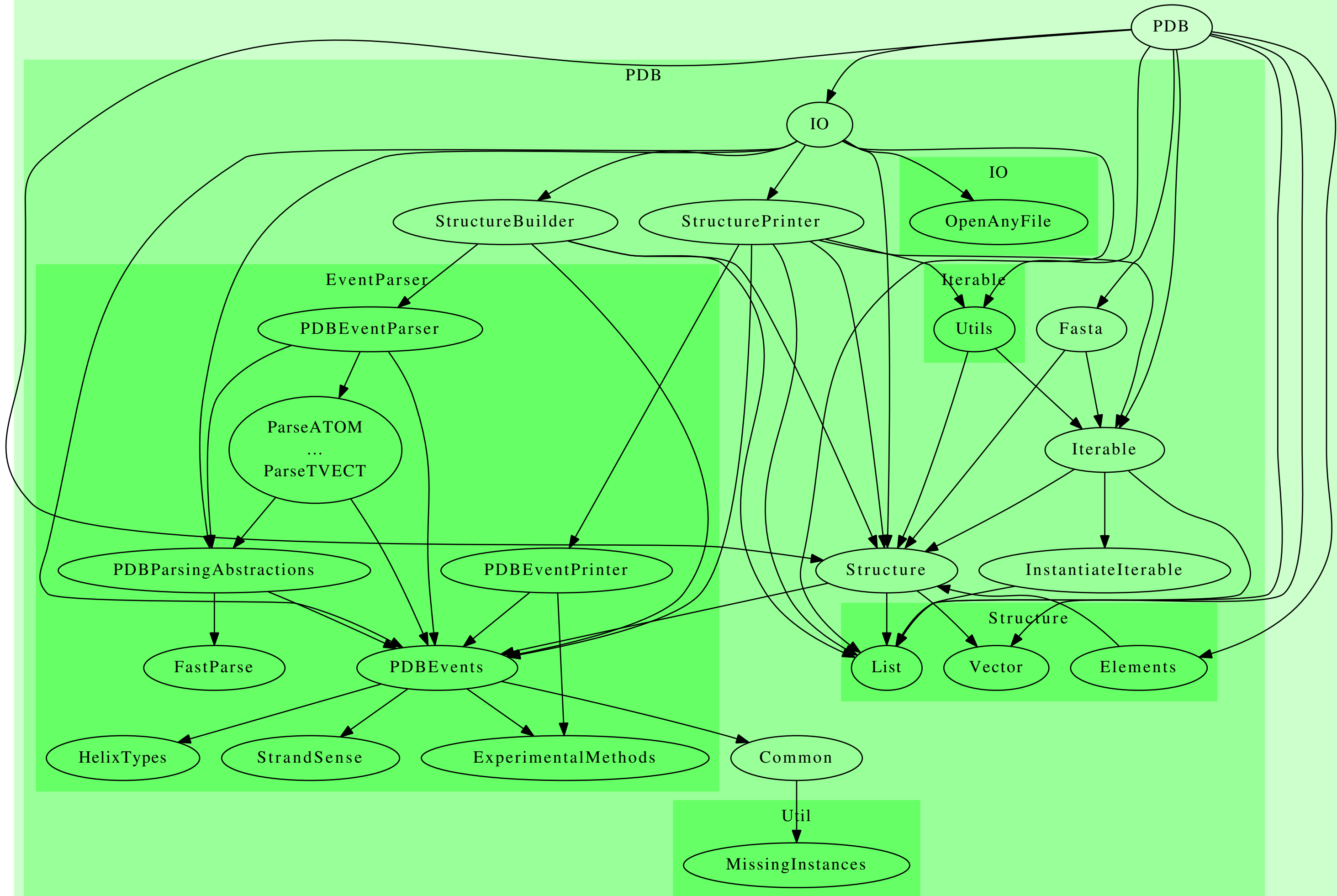

Supplement: Additional file 5 — Module diagram.graphmod.svg contains a module diagram illustrating structure of hPDB. [file 1756-0500-6-483-S5.pdf]
